# Publisher Correction: Accurate autocorrelation modeling substantially improves fMRI reliability

**DOI:** 10.1038/s41467-019-09619-7

**Published:** 2019-03-29

**Authors:** Wiktor Olszowy, John Aston, Catarina Rua, Guy B. Williams

**Affiliations:** 1 Wolfson Brain Imaging Centre, Department of Clinical Neurosciences, University of Cambridge, CB2 0QQ Cambridge, UK; 2 Laboratory of Research in Neuroimaging (LREN), Department of Clinical Neurosciences, CHUV, University of Lausanne, 1011 Lausanne, Switzerland; 3 Statistical Laboratory, Department of Pure Mathematics and Mathematical Statistics, University of Cambridge, CB3 0WB Cambridge, UK

Correction to: *Nature Communications* 10.1038/s41467-019-09230-w, published online 21 March 2019

The original HTML version of this Article had an incorrect Published online date of 25 December 2019; it should have been 21 March 2019. This has been corrected in the HTML version of the Article. The PDF version was correct from the time of publication.

