# Supplementary material for: Accurate autocorrelation modeling substantially improves fMRI reliability
Source: Nat Commun. 2019 Mar 21;10:1220. doi: 10.1038/s41467-019-09230-w (PMC6428826; doi:10.1038/s41467-019-09230-w)
Supplement: Supplementary file 1 — Supplementary Information [file 41467_2019_9230_MOESM1_ESM.pdf]

Accurate autocorrelation modeling substantially improves fMRI reliability

Olszowy et al.

SUPPLEMENTARY INFORMATION

Simulation details

One rest dataset consisted of simulated data generated with the `neuRosim` package in `R`. We used it to simulate 100 resting state scans. The `neuRosim` simulations account for white noise, temporal noise, low-frequency scanner-induced noise, physiological noise, task-related noise and spatial noise. Spatial noise captures spatial relationships in the data: that time series from voxels next to each other tend to be similar. The user specifies the weights of different noises. We arbitrarily chose a weight of 25% corresponding to white noise, a weight of 50% corresponding to temporal noise and a weight of 25% corresponding to spatial noise. For several other tested weights, we could not detect significant activation in any of the 100 simulated scans. `neuRosim` provides AR(m) models to account for temporal autocorrelation. The same model, i.e. with the same parameters, is used for each voxel. We decided to generate the temporally autocorrelated noise with the help of an AR(1) model. For the simulation procedure, a 3-dimensional baseline image must be provided by the user. The voxel-wise means in the simulated scans are equal to this baseline image. We chose a subject from the "FCP Beijing" dataset, subject ID "sub98617", as the baseline subject. The baseline image used for the simulation was the average of the real scan over time. Scanning parameters are shown in Table 1. The number of time points was also chosen as in "FCP Beijing". For the real "FCP Beijing" scan, we arbitrarily chose a cuboidal region of interest, where we calculated the average parameter of voxel-wise AR(1) models. In the simulation procedure it was not possible to directly use the AR(1) parameter from the real "FCP Beijing" scan, as white noise and spatial noise influence the effective value of the parameter of the AR(1) model. That is why we found a parameter for the `neuRosim`'s AR(1) model so that the resulting average AR(1) parameter in the simulated scans in the same cuboidal region of interest was very similar.

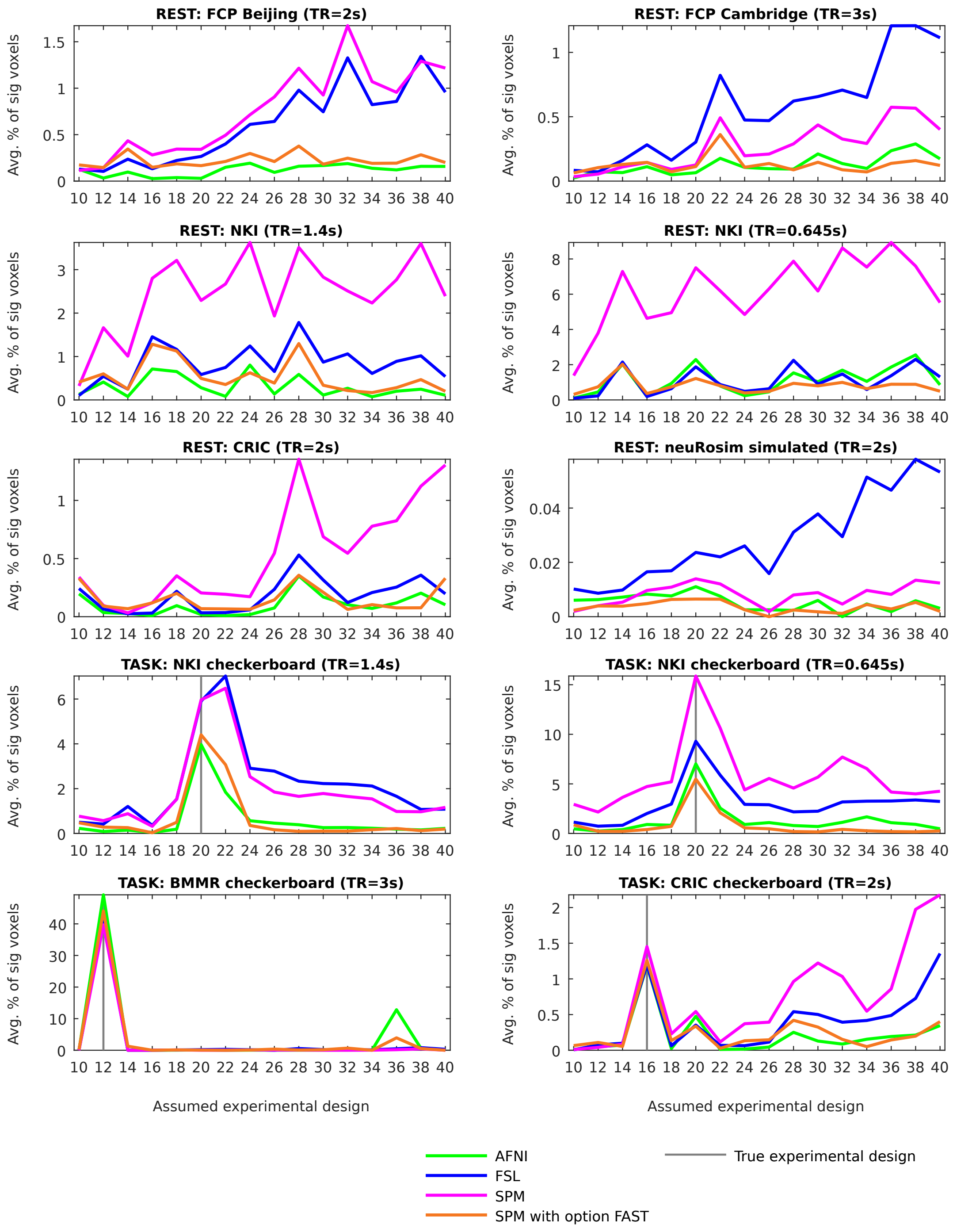

Supplementary Figure 1: Average percentage of significant voxels across subjects for different packages. x-axis shows the assumed designs, e.g. "10" refers to the boxcar design of 10s of rest followed by 10s of stimulus presentation. Scans were spatially smoothed with FWHM of 8 mm. Resting state data was used as null data. Thus, a low percentage of significant voxels was a desirable outcome, as it was suggesting high specificity. Task data with assumed wrong designs was used as null data too. Thus, large positive differences between the true design and the wrong designs were a desirable outcome.

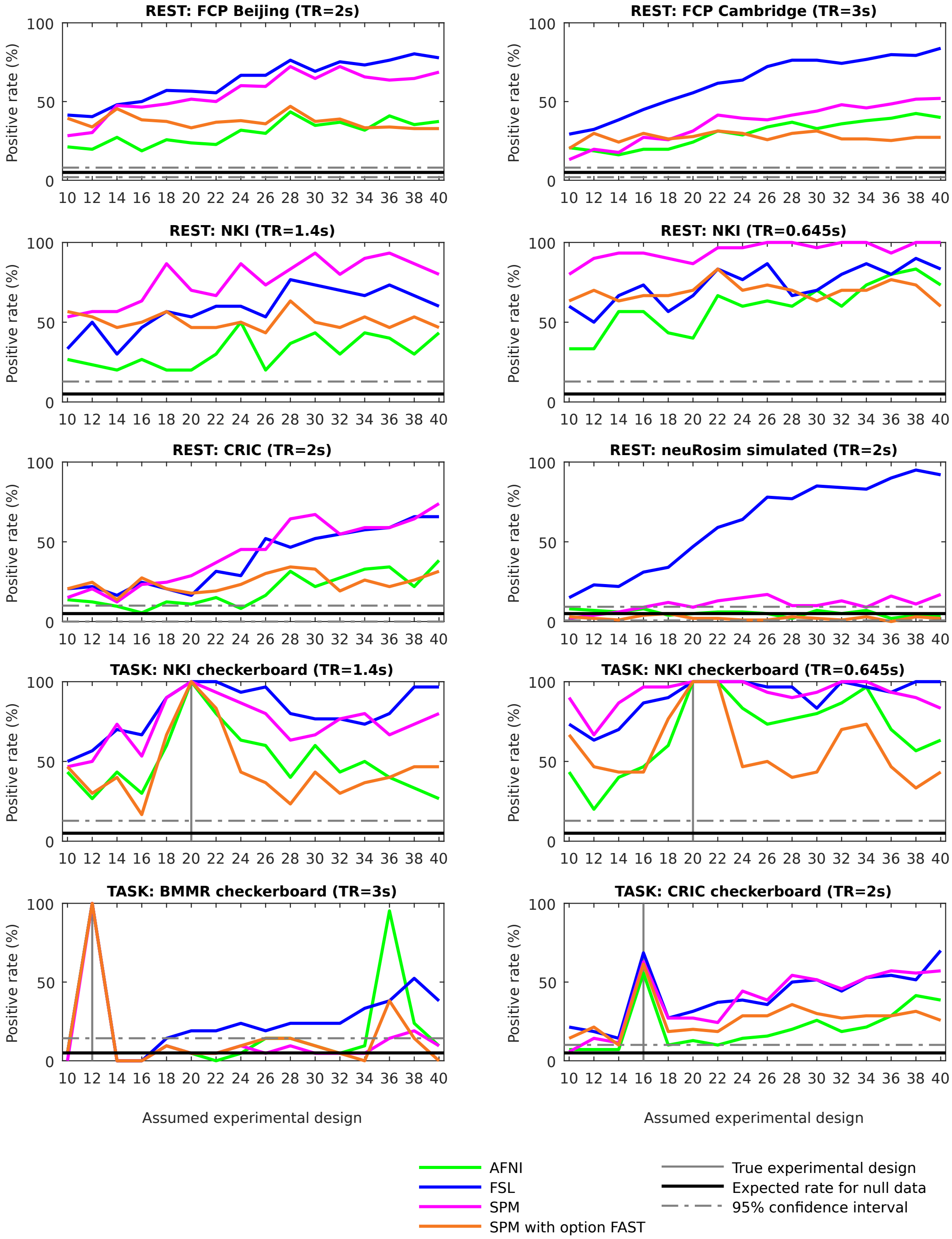

Supplementary Figure 2: Positive rate for different packages. x-axis shows the assumed designs, e.g. "10" refers to the boxcar design of 10s of rest followed by 10s of stimulus presentation. Scans were spatially smoothed with FWHM of **4 mm**. For null data, the positive rate is the familywise error rate. AFNI and `FAST` had the highest specificity.

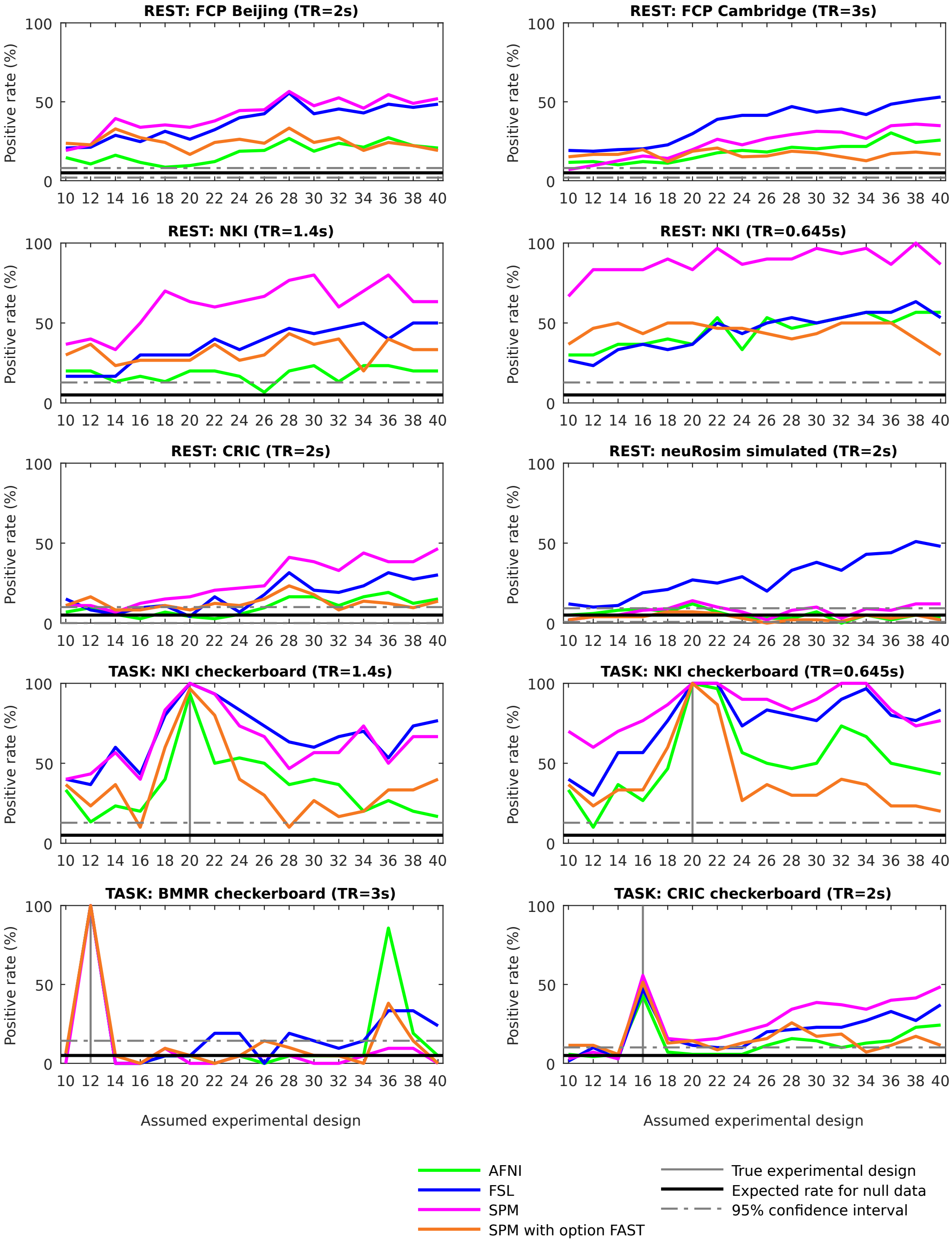

Supplementary Figure 3: Positive rate for different packages. x-axis shows the assumed designs, e.g. "10" refers to the boxcar design of 10s of rest followed by 10s of stimulus presentation. Scans were spatially smoothed with FWHM of **8 mm**. For null data, the positive rate is the familywise error rate. AFNI and `FAST` had the highest specificity.

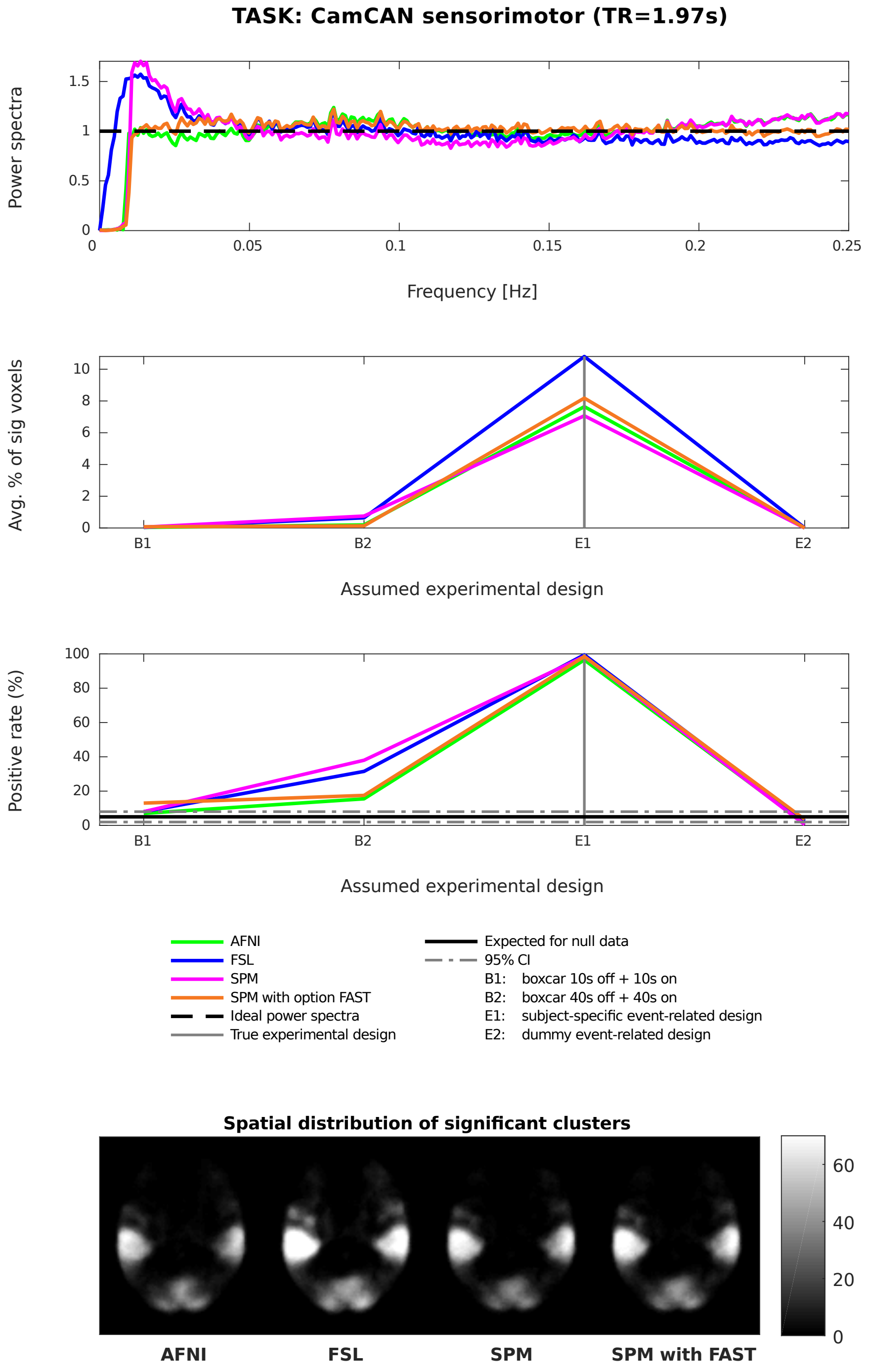

Supplementary Figure 4: Differences between AFNI, FSL, SPM and `FAST` for a task dataset where the design was an event-related design ("CamCAN sensorimotor"). From top to bottom: (1) power spectra of the GLM residuals in native space averaged across brain voxels and across subjects for the assumed true design ("E1"), (2) average percentage of significant voxels for three wrong designs and the true design, (3) positive rate for the same four designs, and (4) spatial distribution of significant clusters for the assumed true design ("E1") on an exemplary MNI axial slice. Scans were spatially smoothed with FWHM of 8 mm.

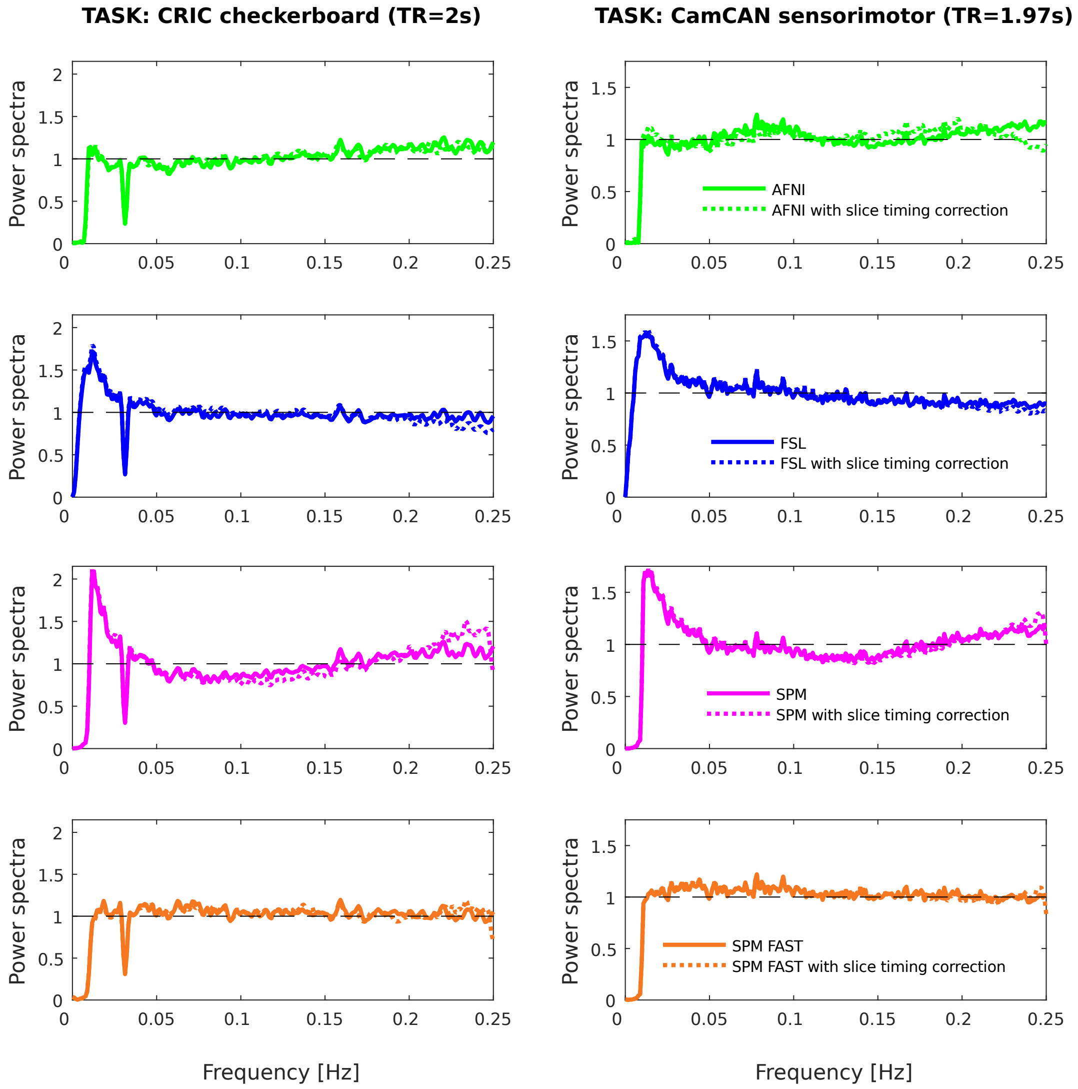

Supplementary Figure 5: Power spectra of the GLM residuals in native space averaged across brain voxels and across subjects for the assumed true designs. Slice timing correction changed the power spectra in a very limited way. Scans were spatially smoothed with FWHM of 8 mm.

CRIC checkerboard (TR=2s)

Pre-whitening	No slice timing correction	Slice timing correction
AFNI	1.19%	1.08%
FSL	1.20%	1.24%
SPM	1.45%	1.35%
SPM with option FAST	1.26%	1.12%

CamCAN sensorimotor (TR=1.97s)

Pre-whitening	No slice timing correction	Slice timing correction
AFNI	7.64%	13.45%
FSL	10.80%	10.68%
SPM	7.07%	6.69%
SPM with option FAST	8.18%	7.78%

Supplementary Table 1: Average percentage of significant voxels across subjects for different packages. Results without slice timing correction are compared to results with slice timing correction. For each dataset, the true design was assumed. Scans were spatially smoothed with FWHM of 8 mm.

**Pre-whitening**	**FWER for summary statistic**	**FWER for mixed effects**
AFNI	15.72%	29.56%
FSL	9.43%	17.61%
SPM	11.95%	18.87%
SPM with option FAST	8.81%	16.35%

Supplementary Table 2: Familywise error rate (FWER) for the SPM's summary statistic model and for the AFNI's mixed effects model (`3dMEMA`) following the use of noise models from AFNI, FSL, SPM and `FAST`. FWER was estimated as the number of null data group analyses with any significant result, divided by the number of null data group analyses (159 for each of the 8 combinations of the group analysis type and of the pre-whitening). Scans were spatially smoothed with FWHM of 8 mm.
